# Renal Allograft Function and the Tacrolimus C/D Ratio: Insights from a Prospective Study on MeltDose Tacrolimus

**DOI:** 10.3390/jcm13206241

**Published:** 2024-10-19

**Authors:** Alicja Dębska-Ślizień, Izabella Kuźmiuk-Glembin, Roman Hożejowski, Dorota Kamińska, Magdalena Krajewska, Anna Zawiasa-Bryszewska, Ilona Kurnatowska, Katarzyna Smykał-Jankowiak, Maciej Głyda, Lidia Kozioł, Marek Karczewski, Kazimierz Ciechanowski, Ewa Kwiatkowska

**Affiliations:** 1 Department of Nephrology, Transplantology and Internal Diseases, Medical University of Gdansk, 80-952 Gdansk, Poland; adeb@gumed.edu.pl (A.D.-Ś.); iza_kuzmiuk@gumed.edu.pl (I.K.-G.); 2 Medical Department, Chiesi Poland Sp. z o.o., 02-305 Warsaw, Poland; 3 Faculty of Medicine, Wroclaw University of Science and Technology, 51-377 Wroclaw, Poland; 4 Department of Internal Diseases and Transplant Nephrology, Medical University of Lodz, 90-153 Lodz, Polandkuril@wp.pl (I.K.); 5 Department of Transplantology, Surgery and Urology, District Hospital, 60-479 Poznan, Poland; 6 Collegium Medicum in Bydgoszcz, Nicolaus Copernicus University, 85-067 Bydgoszcz, Poland; 7 Department of General and Transplant Surgery, Poznan University of Medical Sciences, 60-352 Poznan, Poland; 8 Department of Nephrology, Transplantology and Internal Medicine, Pomeranian Medical University, 70-111 Szczecin, Poland

**Keywords:** kidney transplantation, tacrolimus, MeltDose, LCPT, C/D ratio, pharmacokinetics, glomerular filtration rate

## Abstract

**Background**: The tacrolimus concentration-to-dose (C/D) ratio is valuable for optimizing nephrotoxicity-related renal outcomes. Prospective data on the C/D ratio in kidney transplant recipients newly treated with MeltDose tacrolimus are limited. We analyzed the C/D ratio pattern of MeltDose tacrolimus and its effect on posttransplant renal function, comparing it with the literature data on immediate-release tacrolimus (IR-Tac). **Methods**: In total, 101 adult kidney transplant recipients on a standard immunosuppressive regimen including MeltDose tacrolimus were enrolled in this prospective, multicenter cohort study and followed for 12 months. The C/D ratio classified patients as fast, intermediate, or slow metabolizers. Renal function was assessed via the estimated glomerular filtration rate (eGFR). MeltDose tacrolimus data were compared with previous IR-Tac data by bootstrapping. **Results**: The cohort exhibited a right-skewed C/D ratio distribution with a mean of 2.12 ng/mL × 1/mg, which was significantly greater than the 1.29 mean for IR-Tac (*p* < 0.001). Compared with fast metabolizers, slow metabolizers of MeltDose tacrolimus experienced greater eGFR gains at 6 months post-transplantation (median +7.9 vs. −3.6 mL/min; *p* = 0.005). A Bayesian linear mixed-effects model predicting the eGFR at month 12 identified the baseline eGFR, time from transplant, body mass index, and log-transformed C/D ratio as significant variables. A one-unit increase in the log-transformed C/D ratio corresponded to an approximate increase of 4.5 mL/min in the eGFR at month 12. **Conclusions**: Slow metabolizers of MeltDose tacrolimus had significantly better renal function outcomes than fast metabolizers. MeltDose tacrolimus is associated with slower metabolism than is IR-Tac, as evidenced by its higher C/D ratios.

## 1. Introduction

Tacrolimus plays a pivotal role in the immunosuppressive protocols used in kidney transplantation. However, owing to its narrow therapeutic index and considerable variability in individual pharmacokinetics, tacrolimus blood concentrations can fluctuate significantly, increasing the risk of adverse effects, including nephrotoxicity [1,2]. Elevated tacrolimus levels can cause nephrotoxicity, whereas efforts to lower these levels to mitigate the risk can increase the likelihood of graft rejection.

Thölking and colleagues proposed a simple clinical tool to optimize renal outcomes related to nephrotoxicity [3]. They calculated the ratio of the tacrolimus trough blood concentration to the corresponding tacrolimus dose, referred to as the C/D ratio, in kidney transplant patients treated with immediate-release tacrolimus (IR-Tac). A high C/D ratio indicates slow tacrolimus metabolism, whereas fast metabolizers exhibit a lower C/D ratio. Thölking et al. demonstrated improved renal function in slow metabolizers at all assessed time points after kidney transplantation.

Envarsus^®^ (also known by the acronym LCPT) is an extended-release formulation of tacrolimus produced with MeltDose technology that has a significantly different pharmacokinetic profile than IR-Tac, featuring a more stable blood concentration over time and lower peak concentrations [4]. Detailed data on the distribution of the concentration-to-dose (C/D) ratio from prospective studies of kidney transplant recipients newly treated with MeltDose tacrolimus are still emerging. Most evidence either focuses on conversions from other tacrolimus formulations to MeltDose tacrolimus [5,6,7] or comes from retrospective analyses [8,9].

The goal of this study was to analyze the C/D ratio distribution in kidney transplant recipients newly started on MeltDose tacrolimus and to investigate its impact on renal graft function. Additionally, we examined whether the C/D ratio follows a pattern similar to that reported in the literature for IR-Tac.

## 2. Materials and Methods

### 2.1. Study Design and Patient Enrollment

This prospective cohort study was conducted from February 2020 to May 2023 and enrolled patients from six transplant centers in Poland. Eligible participants were adults who were scheduled to undergo kidney transplantation from deceased donors after brain death or living donors and to initiate a standard immunosuppressive regimen comprising tacrolimus, mycophenolate, and glucocorticosteroids. Depending on immunological risk and in accordance with the study center protocols, patients could receive induction therapy with either basiliximab or antithymocyte globulin.

The participants either started MeltDose tacrolimus at a daily dose of 0.17 mg/kg body weight from the day of transplantation (day 1) or were switched from immediate-release tacrolimus (IR-Tac) to MeltDose tacrolimus by day 8 at the latest. In cases of conversion, a ratio of 1:0.7 (mg/mg) was applied, with further dosages adjusted to achieve target tacrolimus levels. These target levels were consistent for all patients: 12–15 ng/mL during the first month, 8–12 ng/mL thereafter, and 5–10 ng/mL from month 3 to month 12, in line with the guidelines of the Polish Transplantation Society [10].

### 2.2. Follow-Up and Study Outcomes

Patients were followed-up for 12 months, with a baseline visit on day 14 and follow-up visits at months 1, 3, 6, and 12 after transplantation. Tacrolimus trough concentrations were measured in whole-blood samples collected after an overnight fast and prior to the morning tacrolimus dose. The primary study endpoint was the MeltDose tacrolimus C/D ratio, which was calculated as the mean of the C/D ratios at months 1, 3, and 6, as proposed by Thölking in his pivotal study [3]. The mean C/D ratio, which was split into tertiles, was used to classify patients as fast, intermediate, or slow metabolizers. The main renal function outcome was the change in the estimated glomerular filtration rate (eGFR) over time, which was calculated with the CKD-EPI creatinine equation. Serum creatinine levels were determined using photometric method on the Alinity ci-series analyzer (Abbott Diagnostics, Wiesbaden, Germany). The baseline eGFR was set at month 1.

### 2.3. Statistical Analysis

The distribution of the C/D ratio was described as the mean, range, and skewness. For statistical testing, the mean C/D ratio of MeltDose tacrolimus was compared with that of IR-Tac from a pivotal study [3] using the bootstrap method with 1000 resamplings. Bootstrapping employs machine learning algorithms for resampling the initial data (in this case, the C/D ratio for IR-Tac) to derive estimates of standard errors and confidence intervals [11].

Changes in the eGFR were compared between slow metabolizers and fast metabolizers using the Mann–Whitney U test.

A Bayesian linear mixed-effects model, including baseline characteristics and the log-transformed C/D ratio as explanatory variables, was used to analyze the effect of the C/D ratio on the eGFR at month 12. Selection of the final model was based on the Watanabe–Akaike information criterion. Changes in the tacrolimus dose, trough concentration (C0), and corresponding C/D ratios were analyzed with Friedman’s ANOVA with Dunn’s post hoc tests. Two-sided *p*-values < 0.05 were considered significant. Statistical analyses were conducted with R software (version 4.3.0, Foundation for Statistical Computing, Vienna, Austria).

## 3. Results

**Figure 1:**
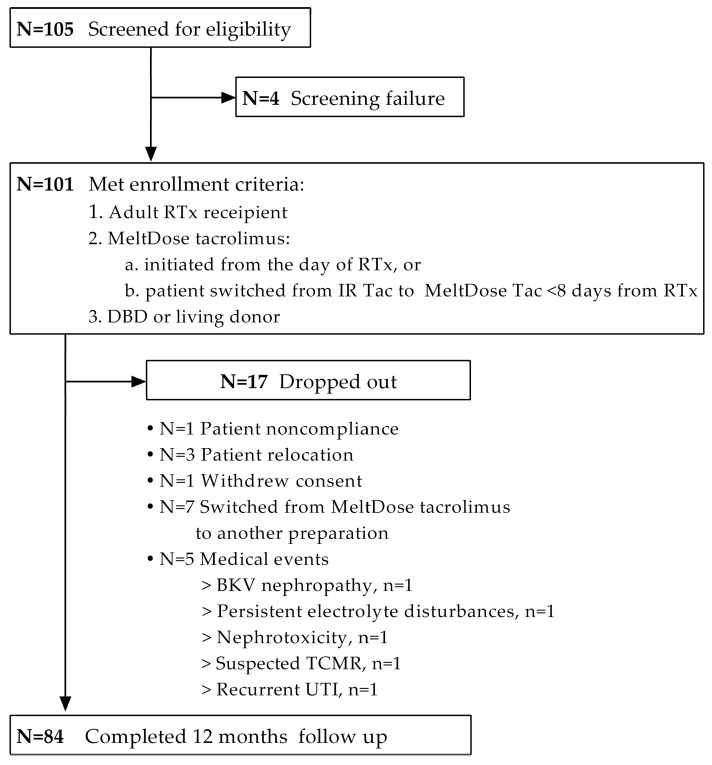
Disposition of the study participants. Abbreviations: RTx = renal transplant; Tac = tacrolimus; IR-Tac = immediate-release tacrolimus; DBD = donation after brainstem death; TCMR = T-cell-mediated rejection; UTI = urinary tract infection.

A total of 101 patients were enrolled in this prospective cohort study, all of whom were of White ethnicity. The median (IQR) follow-up time was 11.7 (10.9–12.2) months, and a 12-month follow-up was available for 84 patients. The detailed distribution of the study patients is shown in Figure 1.

### 3.1. Metabolizer Classification and the C/D Ratio

**Figure 2:**
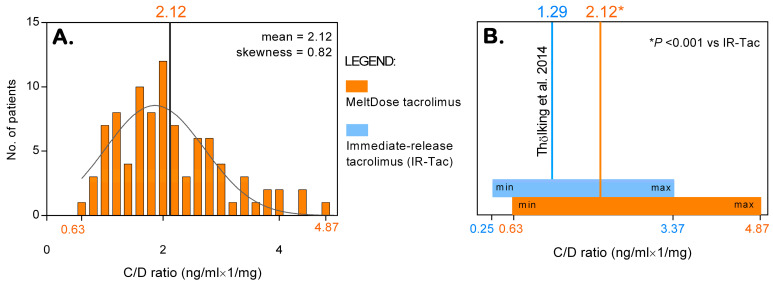
Distribution of the C/D ratio for MeltDose tacrolimus (**A**) and comparison with the distribution for immediate-release tacrolimus (**B**).

The C/D ratio increased significantly over time, with a mean of 1.67 ng/mL × 1/mg at month 1 and increasing to 2.84 ng/mL × 1/mg at month 12 (*p* < 0.001). Data from 93 patients with complete C/D ratio measurements at months 1, 3, and 6 allowed for the calculation of the average C/D ratio with Thölking’s methodology. The distribution of the average C/D ratio was right-skewed (skewness = 0.82), with a mean of 2.12 ng/mL × 1/mg (95% CI: 1.93–2.31), which was significantly greater (*p* < 0.001) than the mean C/D ratio of 1.29 reported for IR-Tac in Thölking’s study [3] (see Figure 2).

The study participants were classified into tertiles on the basis of their C/D ratios: fast metabolizers (C/D ratio <1.63 ng/mL × 1/mg), intermediate metabolizers (1.63–2.34 ng/mL × 1/mg), and slow metabolizers (>2.34 ng/mL × 1/mg). Among the cohort, 34% (*n* = 32) were classified as slow metabolizers, while intermediate (*n* = 31) and fast metabolizers (*n* = 30) each represented 33%. The baseline characteristics were comparable across the metabolizer subgroups (Table 1).

### 3.2. Tacrolimus Dose and Trough Concentration

**Figure 3:**
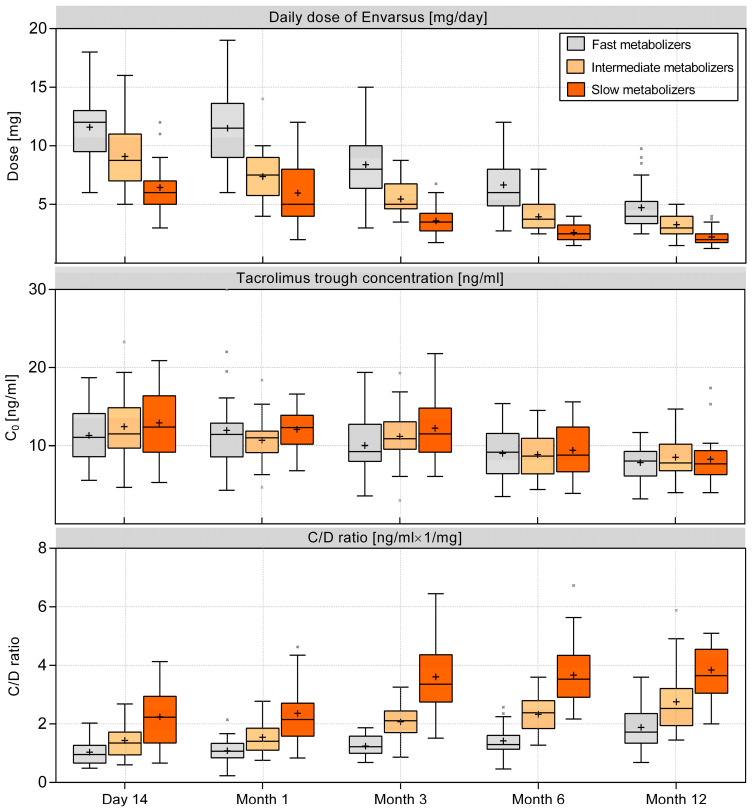
Tacrolimus doses, trough concentrations, and C/D ratios over the 12-month follow-up period, stratified by metabolizer subgroups. The boxes depict interquartile ranges, with horizontal lines representing the medians and crosses representing the means. Points outside the whiskers are identified as Tukey outliers.

Tacrolimus trough concentrations (C0) remained stable from day 14 to month 3, with median C0 levels ranging between 10.6 and 11.6 ng/mL. Fast metabolizers required higher daily doses (median 11.5 mg at month 1) compared to slow metabolizers (median 5 mg at month 1; *p* < 0.001). From month 6 to month 12, lower C0 levels were maintained (median 8.8 ng/mL at month 6 and 7.8 ng/mL at month 12), with fast metabolizers continuing to need higher daily doses (median 4 mg vs. 2 mg at month 12; *p* < 0.001) (Figure 3).

### 3.3. Renal Function Outcomes

**Figure 4:**
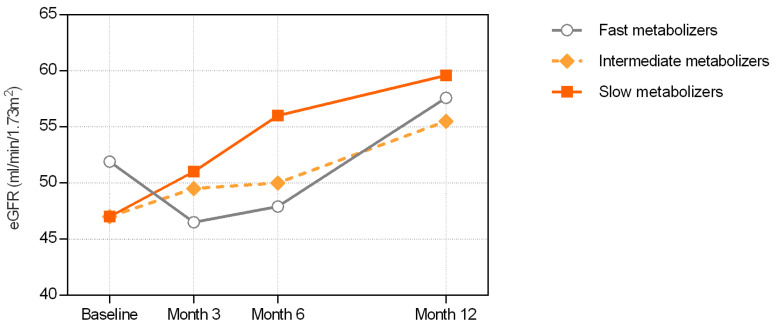
Renal function during the study period. Median values of CKD-EPI eGFR stratified by metabolizer group.

The cohort exhibited stable eGFR levels in the first 3 months, followed by an increasing trend until month 12. Slow and intermediate metabolizers showed a consistent improvement in eGFR (Figure 4). In slow metabolizers, the median (IQR) eGFR increased from a baseline value of 47 (41–55.2) to 59.6 (42.5–69) mL/min at month 12. Similarly, intermediate metabolizers exhibited an increase in eGFR from 47 (35.6–60.8) at baseline to 55.5 (46.9–65.8) mL/min at month 12.

In contrast, fast metabolizers exhibited a different pattern. Despite having the highest median eGFR at baseline among all groups at 51.9 (38.3–64.5) mL/min, the fast metabolizers presented a mostly declining trend in the subsequent months, ultimately reaching a comparable level of 57.6 (45.9–70.5) mL/min only at month 12 (Figure 4).

### 3.4. Comparison of Renal Function Recovery between Fast and Slow Metabolizers

Compared with fast metabolizers, slow metabolizers showed superior renal function recovery after transplantation. At month 6, slow metabolizers had a median (IQR) eGFR increase of 7.9 (2.2–12.7) mL/min vs. baseline, which contrasted significantly with a decrease of –3.6 (−10.2–4.9) mL/min in fast metabolizers (*p* = 0.005).

### 3.5. Bayesian Model for Predicting Renal Function

**Figure 5:**
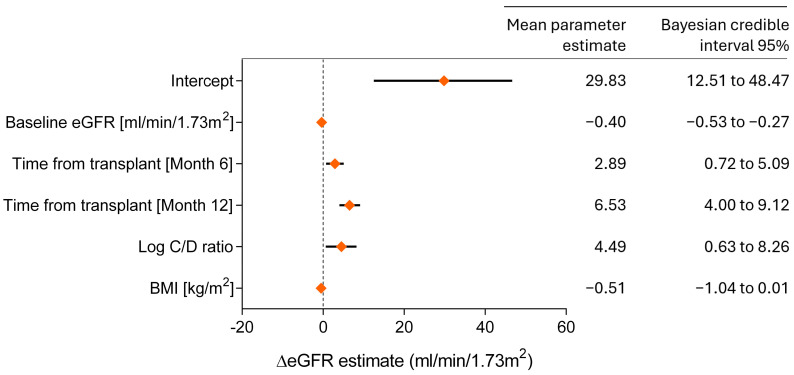
Predictors of the change in eGFR at month 12 (compared with baseline)—Bayesian linear mixed-effects model. The C/D ratio was logarithmically transformed to adjust for skewness in the distribution.

In the final Bayesian mixed-effects model predicting eGFR at month 12, the key explanatory variables were baseline eGFR, time since transplant, body mass index (BMI), and the log-transformed C/D ratio (log C/D ratio) (Figure 5). The mean estimate for the log C/D ratio was 4.49, indicating that, all else being equal, a hypothetical patient with a log C/D ratio higher by one unit would at month 12 have an eGFR approximately 4.5 mL/min higher than that of another patient with the same BMI and baseline eGFR.

### 3.6. Graft Rejections and Viral Infections

T-cell-mediated rejection occurred in one intermediate metabolizer who withdrew from the study, received intravenous methylprednisolone pulses, and was converted to IR-Tac. Another intermediate metabolizer experienced humoral rejection, which was successfully treated with plasmapheresis, bortezomib, and intravenous immunoglobulin G.

BK virus replication was confirmed in blood specimens from 7 out of 101 patients (6.9%), including 4 fast tacrolimus metabolizers (13.3%), 2 intermediate metabolizers (6.5%), and 1 slow metabolizer (3.1%)

## 4. Discussion

This study provides insights into the pharmacokinetic profile and renal function outcomes associated with MeltDose tacrolimus. Specifically, we present extensive information on the distribution of the C/D ratio in de novo-treated kidney transplant recipients. This aspect is particularly important, as existing evidence is still emerging.

Data on the C/D ratio in de novo kidney transplant recipients are available from a post hoc analysis of a randomized comparative trial by Suwelack et al., which evaluated the C/D ratios of MeltDose tacrolimus versus IR-Tac [12]. However, those authors used a methodology that differed from Thölking’s original model, calculating the C/D ratio on day 30 after transplantation. Although the publication did not report mean or median C/D values, it did provide tertile intervals corresponding to tacrolimus metabolism rates. Analysis of these ranges revealed a shift toward higher values in the MeltDose tacrolimus group than in the IR-Tac group, suggesting a predominance of slower metabolism. Consistent with this result, a comparison of the C/D ratio distribution in our cohort with the literature data on IR-Tac from Thölking’s pivotal study [3] revealed that the mean C/D ratio for MeltDose tacrolimus was significantly greater than that of IR-Tac, and this indicated an overall trend toward slower metabolism. Our analysis, along with the aforementioned findings, highlights notable differences in absorption, metabolism, and elimination processes between the tacrolimus formulations, with MeltDose tacrolimus achieving higher steady-state concentrations relative to the administered dose.

Another study by Fernandez-Rivera et al. reported the MeltDose tacrolimus C/D ratios at consecutive timepoints over a 6-month follow-up [13]. Their data were obtained from a prospective comparative study involving MeltDose tacrolimus (Envarsus, LCPT) and prolonged-release tacrolimus (Advagraf, PR-Tac) in de novo kidney transplant recipients. Similar to the previously mentioned study, the results show that the C/D ratios for the LCPT group were consistently higher than those for the PR-Tac group throughout the entire follow-up period.

The concept of stratifying patients into fast, intermediate, and slow metabolizers on the basis of the C/D ratio is a clinically useful tool and an important indicator of the drug pharmacokinetics. In this context, it is surprising that the median C/D ratio for prolonged-release tacrolimus (PR-Tac), a once-daily formulation that was different from MeltDose tacrolimus and investigated by Thölking, was 1.30 ng/mL × 1/mg [14]. This value is very similar to the value of 1.29 ng/mL × 1/mg reported for IR-Tac by the same research group [3] despite the entirely different pharmacokinetic profiles. Other renal transplant studies, such as the one by Nowicka et al. [15], report a median C/D ratio of 1.51 ng/mL × 1/mg in a mixed population using both immediate- and prolonged-release tacrolimus, which is close to Thölking’s IR-Tac result. Similarly, Kwiatkowska et al. [16] reported a median C/D ratio of 1.68 ng/mL × 1/mg derived from a population using various formulations of tacrolimus. All the aforementioned average tacrolimus C/D ratios were lower than the mean of 2.12 ng/mL × 1/mg reported in our cohort. The higher C/D ratio observed in our study than in reports on other tacrolimus formulations reflects a tendency toward slower metabolism, which may be attributed to the unique characteristics of the drug formulation. The MeltDose technology enables the active substance to be absorbed over a longer length of the gastrointestinal tract, including the distal section of the small intestine and the large intestine [17]. Because of the lower expression of CYP3A5 in these distal portions, the bioavailability of MeltDose tacrolimus is better than that of IR-Tac [6].

However, the differences in C/D ratios may arise not only from the use of different tacrolimus formulations but also from the timing of the measurements. Our study reports the C/D ratio averaged at 1, 3, and 6 months post transplantation, which aligns with the original study by Thölking [3]. In contrast, Nowicka et al. [15] reported the median C/D ratio at month 6, whereas Thölking’s 2021 study [18] reported the median C/D ratio at month 3. The TOMATO study, which involved over 1000 patients in France who were treated with both IR-Tac and PR-Tac, provided C/D ratios for months 3, 6, and 12 and reported stabilization of the C/D ratio between 6 and 12 months after transplantation [19]. This observation aligns with our study, which showed that the C/D ratio tends to increase after therapy initiation but stabilizes around 3–6 months and remains consistent thereafter. This suggests that the C/D ratio measured after at least 6 months accurately reflects the patient’s long-term metabolic profile. However, the Thölking methodology, which also considers the initial months, seems justified. This approach “corrects” the C/D ratio downwards due to lower initial C/D values and alerts physicians to closely monitor patients who tend to exhibit fast tacrolimus metabolism. This may be especially useful in the initial period after transplantation when graft function is still stabilizing.

Genetic factors also play a role in the variability of C/D ratios. Several studies have documented the association between the CYP3A5 genotype and the response to tacrolimus, with these genetic variations influencing both tacrolimus-related adverse effects and graft rejection [20,21,22]. To reach the target drug concentration necessary to prevent rejection, patients who carry the CYP3A5*1/*1 and *1/*3 genotypes (expressors) require greater dosages. Consequently, these individuals have lower C/D ratios. Conversely, individuals with the CYP3A5*3/*3 genotype, i.e., nonexpressors, have high C/D ratios (slow metabolizers) and are recommended lower tacrolimus doses to avoid adverse drug reactions [23]. There is evidence to suggest that CYP3A5 genotype status and a low C/D ratio are not fully overlapping factors [24]. Consequently, gene polymorphism studies could help identify vulnerable patients at risk for tacrolimus-induced nephrotoxicity. On the other hand, long-term follow-up from the Tactique study showed that adjusting tacrolimus dosage based on CYP3A5 genotype did not significantly improve graft survival, reduce rejection rates, or lower adverse events in kidney transplant patients [25]. These findings, along with the considerable costs and limited accessibility of genetic testing, remain significant barriers to its widespread adoption in routine clinical practice. Future approaches should incorporate a comprehensive risk assessment model that integrates multiple demographic, clinical, and genetic factors rather than focusing solely on single pharmacogenetic markers like CYP3A5.

Concomitant medication and dietary interactions are another source of C/D variability. Several substances can induce or inhibit cytochrome P450 enzymes, significantly increasing (e.g., ketoconazole and erythromycin) or decreasing (e.g., rifampicin) tacrolimus levels [26]. Prednisone is also known to affect tacrolimus metabolism, and an increase in the C/D ratio would be expected as the prednisone dose is reduced after transplantation [27].

In our study, categorizing patients into fast, intermediate, and slow metabolizers on the basis of their C/D ratios revealed significant differences in renal function recovery. Compared with fast metabolizers, slow metabolizers of MeltDose tacrolimus presented superior renal function at 6 months post transplantation. Additionally, the Bayesian linear mixed-effects model revealed that the C/D ratio was a significant predictor of renal function at month 12, indicating that higher C/D values are associated with greater increases in the eGFR. These findings align with previous studies suggesting that higher C/D ratios, indicative of slower tacrolimus metabolism, are beneficial for renal graft function. A pivotal study conducted by Thölking in 2014 reported improved renal function in slow metabolizers of IR-Tac [3]. The same investigators found that liver transplant recipients classified as fast tacrolimus metabolizers had worse renal function at the 3-year follow-up [28]. Compared with slow metabolizers, fast metabolizers also had significantly poorer graft function in the previously mentioned studies, including Nowicka et al.’s cohort [15].

An intriguing observation from our study was the detection of BK virus in four tacrolimus fast metabolizers (13.3%) but in only two intermediate metabolizers (6.5%) and one slow metabolizer (3.1%). Although the small number of cases precluded statistical significance, the data suggest a higher prevalence of BK virus infections among individuals with fast tacrolimus metabolism. Previous research also indicated that fast tacrolimus metabolizers are more prone to BK virus infections. Nowicka et al. reported BK nephropathy exclusively in patients with fast metabolism of tacrolimus (two cases) [15]. In contrast, von Samson-Himmelstjerna et al. did not find a correlation between the rate of tacrolimus metabolism and the frequency of BK virus infections, although they reported BK viremia in 20.1% of patients [29]. An in vitro study revealed that tacrolimus directly stimulates BK virus replication by binding to FKBP-12 [30], which may account for the increased susceptibility to BK infection among fast metabolizers, as they receive higher doses of tacrolimus.

Despite the strengths of this study, including its prospective design and multicenter approach, there are limitations to consider. The relatively small sample size and single-country setting can limit the applicability of the findings to larger populations. Importantly, the comparison with IR-Tac is not direct but is based on the literature data from Thölking’s pivotal study [3]. Although this comparison is indirect, it is supported by detailed data on the C/D ratio distribution, which was a major focus of Thölking’s research and was presented comprehensively in his publication.

The results of our study hold practical significance, as they represent a step toward a more personalized approach in immunosuppressive therapy, allowing for the simple and cost-free identification of patients at higher risk for tacrolimus toxicity. These patients could benefit from increased monitoring. However, both the findings of this study and the available evidence are not yet sufficient to warrant more radical changes in treatment strategy, such as setting different target ranges of tacrolimus concentrations for slow and fast metabolizers. These modifications require validation of their safety through randomized controlled trials, which should consider not only pharmacokinetic parameters but also other critical factors, such as comorbidities and the recipients’ immunological risk profiles.

In conclusion, the study added to the body of evidence on the importance of monitoring tacrolimus C/D ratio during immunosuppressive therapy in patients after kidney transplantation. While a detailed description of this parameter for MeltDose tacrolimus was provided, we also confirmed that slow tacrolimus metabolism is beneficial for the allograft function. MeltDose tacrolimus shows higher C/D values compared to IR-Tac, indicating tendency to slower metabolism.

## Figures and Tables

**Table 1:** Baseline characteristics of study patients. Metabolic stratification was performed for 93 out of 101 patients. Due to incomplete data, eight patients (7.9%) were not classified. Data are presented as *n* (%), medians (IQR), or means ± SD. Abbreviations: BMI = body mass index; Tac = tacrolimus; PRA = panel-reactive antibodies; MMF = mycophenolate mofetil.

	All Patients	A. Fast Metabolizers	B. Intermediate Metabolizers	C. Slow Metabolizers	*p*-Value A vs. B vs. C	*p*-Value A vs. C
N	101	30/101	31/101	32/101	—	—
%	(100%)	(29.7%)	(30.7%)	(31.7%)		
**Demographics and allograft function**						
Age, years	46.8 ± 12.3	44.9 ± 11.7	45.9 ± 11	49.4 ± 14	0.344	0.197
Sex (male)	50 (49.5%)	14 (46.7%)	16 (51.6%)	16 (50%)	0.925	0.994
BMI (kg/m^2^)	25.3 ± 4	25.1 ± 4.1	25.9 ± 4.2	24.8 ± 3.8	0.791	0.844
Serum creatinine (mg/dL)	1.6 ± 0.6	1.6 ± 0.6	1.6 ± 0.6	1.5 ± 0.4	0.662	0.893
eGFR (mL/min/1.73 m^2^)	47.3 (38–61.6)	51.9 (38.3–64.5)	47 (35.6–60.8)	47 (41–55.2)	0.896	0.690
**Significant comorbidity**						
Arterial hypertension	69 (68.3%)	23 (76.7%)	22 (71%)	16 (50%)	0.065	0.056
Ischemic heart disease	14 (13.8%)	2 (6.7%)	5 (16.1%)	7 (21.9%)	0.217	0.197
Type 2 diabetes mellitus	11 (10.9%)	3 (10%)	2 (6.5%)	6 (18.8%)	0.340	0.475
Type 1 diabetes mellitus	3 (3%)	1 (3.3%)	2 (6.5%)	0 (0%)	—	—
**Etiologies of end-stage renal disease**						
Glomerulonephritis	43 (42.6%)	8 (26.6%)	16 (51.6%)	15 (46.8%)	0.074	0.236
Polycystic kidney disease	14 (13.9%)	7 (23.3%)	3 (9.7%)	4 (12.5%)	0.336	0.329
Tubulointerstitial nephritis	9 (8.9%)	3 (10%)	3 (9.7%)	1 (3.1%)	0.556	0.346
Hypertensive nephropathy	8 (7.9%)	3 (10%)	0 (0%)	5 (15.6%)	0.066	0.709
Diabetic nephropathy	7 (6.9%)	2 (6.7%)	3 (9.7%)	2 (6.2%)	0.894	1.000
Congenital urinary abnormalities	3 (3%)	0 (0%)	1 (3.2%)	0 (0%)	—	—
Other	23 (22.8%)	9 (30%)	6 (19.4%	6 (18.8%)	0.497	0.461
**Previous transplants**	13 (12.9%)	3 (10%)	4 (12.9%)	5 (15.6)	0.926	0.709
**PRA (%)**	5.4 ± 12.4	5.7 ± 12.2	3.7 ± 8.1	6.5 ± 16.1	0.278	0.943
**PRA <5%**	80 (79%)	24 (80%)	25 (81%)	25 (78%)	0.967	1.000
**Donor characteristics**						
Age	48.6 ± 13.2	47.8 ± 11.5	47.1 ± 13.4	48.6 ± 15.1	0.839	0.800
Sex (male)	65 (64.4%)	19 (63.3%)	21 (67.7%)	20 (62.5)	0.898	1.000
Living donor	6 (5.9%)	1 (3.3%)	3 (9.7%)	2 (6.2)	0.778	1.000
**Transplant characteristics**						
HLA antigen mismatches	3 (3–4)	3 (2–4)	3 (3–4)	3 (3–4)	0.344	0.211
Cold ischemia time (h)	14.8 (10.4–21.2)	14.5 (11.3–19.8)	14.3 (10.2–20.8)	14.3 (10.3–19.7)	0.875	0.683
Warm ischemia time (min)	28 (24–33)	30 (25–34)	27 (22–30)	28 (24–35)	0.153	0.498
Delayed graft function	24 (23.8%)	10 (30%)	7 (22.6)	5 (15.6)	0.282	0.141
**Induction therapy** **(basiliximab or thymoglobulin)**	82 (81.2%)	21 (70%)	25 (80.6%)	28 (87.5%)	0.229	0.123
**Maintenance immunosuppression**					0.350	0.254
Tac + MMF + methylprednisolone	68 (67.3%)	22 (73.3%)	21 (67.7%)	21 (67.7%)		
Tac + MMF + prednisone	32 (31.7%)	8 (26.7%)	10 (32.3%)	10 (32.3%)		
Tac + MMF + deflazacort	1 (0.9%)	0 (0%)	0 (0%)	0 (0%)		

## Data Availability

The datasets analyzed during the current study are available from the corresponding author on reasonable request.

## References

[1] Kim H., Han A., Ahn S., Min S.K., Ha J., Min S. (2023). Association of high intra-patient variability in tacrolimus exposure with calcineurin inhibitor nephrotoxicity in kidney transplantation. Sci. Rep..

[2] Gijsen V.M., Madadi P., Dube M.P., Hesselink D.A., Koren G., de Wildt S.N. (2012). Tacrolimus-induced nephrotoxicity and genetic variability: A review. Ann. Transplant..

[3] Thölking G., Fortmann C., Koch R., Gerth H.U., Pabst D., Pavenstädt H., Kabar I., Hüsing A., Wolters H., Reuter S. (2014). The tacrolimus metabolism rate influences renal function after kidney transplantation. PLoS ONE.

[4] Tremblay S., Nigro V., Weinberg J., Woodle E.S., Alloway R.R. (2017). A Steady-State Head-to-Head Pharmacokinetic Comparison of All FK-506 (Tacrolimus) Formulations (ASTCOFF): An Open-Label, Prospective, Randomized, Two-Arm, Three-Period Crossover Study. Am. J. Transplant..

[5] Giral M., Grimbert P., Morin B., Bouvier N., Buchler M., Dantal J., Garrigue V., Bertrand D., Kamar N., Malvezzi P. (2024). Impact of Switching From Immediate- or Prolonged-Release to Once-Daily Extended-Release Tacrolimus (LCPT) on Tremor in Stable Kidney Transplant Recipients: The Observational ELIT Study. Transpl. Int..

[6] Thölking G., Tosun-Koç F., Jehn U., Koch R., Pavenstädt H., Suwelack B., Reuter S. (2022). Improved Kidney Allograft Function after Early Conversion of Fast IR-Tac Metabolizers to LCP-Tac. J. Clin. Med..

[7] Sánchez Fructuoso A., Ruiz J.C., Franco A., Diekmann F., Redondo D., Calviño J., Serra N., Aladrén M.J., Cigarrán S., Manonelles A. (2020). Effectiveness and safety of the conversion to MeltDose® extended-release tacrolimus from other formulations of tacrolimus in stable kidney transplant patients: A retrospective study. Clin. Transplant..

[8] Czarnecka P., Czarnecka K., Baczkowska T., Lagiewska B., Durlik M. (2023). Real-life comparison of efficacy and safety profiles of two prolonged-release tacrolimus formulations in de novo kidney transplant recipients: 24 months of follow-up. PLoS ONE.

[9] Glander P., Waiser J., Kasbohm S., Friedersdorff F., Peters R., Rudolph B., Wu K., Budde K., Liefeldt L. (2018). Bioavailability and costs of once-daily and twice-daily tacrolimus formulations in de novo kidney transplantation. Clin. Transplant..

[10] Durlik M., Danielewicz R. (2021). Zalecenia Dotyczące Leczenia Immunosupresyjnego po Przeszczepieniu Narządów Unaczynionych [Recommendations for Immunosuppressive Treatment after Transplantation of Vascularized Organs].

[11] Carpenter J., Bithell J. (2000). Bootstrap confidence intervals: When, which, what? A practical guide for medical statisticians. Stat. Med..

[12] Suwelack B., Bunnapradist S., Meier-Kriesche U., Stevens D.R., Procaccianti C., Morganti R., Budde K. (2020). Effect of Concentration/Dose Ratio in De Novo Kidney Transplant Recipients Receiving LCP-Tacrolimus or Immediate-Release Tacrolimus: Post Hoc Analysis of a Phase 3 Clinical Trial. Ann. Transplant..

[13] Fernandez Rivera C., Calvo Rodríguez M., Poveda J.L., Pascual J., Crespo M., Gomez G., Cabello Pelegrin S., Paul J., Lauzurica R., Perez Mir M. (2022). Better study. Bioavailability of once-daily tacrolimus formulations used in clinical practice in the management of De Novo kidney transplant recipients: The better study. Clin. Transplant..

[14] Thölking G., Filensky B., Jehn U., Schütte-Nütgen K., Koch R., Kurschat C., Pavenstädt H., Suwelack B., Reuter S., Kuypers D. (2021). Increased renal function decline in fast metabolizers using extended-release tacrolimus after kidney transplantation. Sci. Rep..

[15] Nowicka M., Górska M., Nowicka Z., Edyko K., Edyko P., Wiślicki S., Zawiasa-Bryszewska A., Strzelczyk J., Matych J., Kurnatowska I. (2019). Tacrolimus: Influence of the Posttransplant Concentration/Dose Ratio on Kidney Graft Function in a Two-Year Follow-Up. Kidney Blood Press Res..

[16] Kwiatkowska E., Kwiatkowski S., Wahler F., Gryczman M., Domańki L., Marchelk-Myśliwiec M., Ciechanowski K., Drozd-Dabrowska M. (2019). C/D Ratio in Long-Term Renal Function. Transplant Proc..

[17] Tremblay S., Alloway R.R. (2017). Clinical Evaluation of Modified Release and Immediate Release Tacrolimus Formulations. AAPS J..

[18] Thölking G., Schulte C., Jehn U., Schütte-Nütgen K., Pavenstädt H., Suwelack B., Reuter S. (2021). The Tacrolimus Metabolism Rate and Dyslipidemia after Kidney Transplantation. J. Clin. Med..

[19] Jouve T., Fonrose X., Noble J., Janbon B., Fiard G., Malvezzi P., Stanke-Labesque F., Rostaing L. (2020). The TOMATO Study (Tacrolimus Metabolization in Kidney Transplantation): Impact of the Concentration-Dose Ratio on Death-censored Graft Survival. Transplantation.

[20] Marco D.N., Molina M., Guio A.M., Julian J., Fortuna V., Fabregat-Zaragoza V.L., Salas M.Q., Monge-Escartín I., Riu-Viladoms G., Carcelero E. (2024). Effects of CYP3A5 Genotype on Tacrolimus Pharmacokinetics and Graft-versus-Host Disease Incidence in Allogeneic Hematopoietic Stem Cell Transplantation. Pharmaceuticals.

[21] Du W., Wang X., Zhang D., Zuo X. (2024). Genotype-Guided Model for Prediction of Tacrolimus Initial Dosing After Lung Transplantation. J. Clin. Pharmacol..

[22] Cai L., Ke M., Wang H., Wu W., Lin R., Huang P., Lin C. (2023). Physiologically based pharmacokinetic model combined with reverse dose method to study the nephrotoxic tolerance dose of tacrolimus. Arch. Toxicol..

[23] Chauhan P.M., Hemani R.J., Solanki N.D., Shete N.B., Gang S.D., Konnur A.M., Srivastava R., Pandey S.N. (2023). A systematic review and meta-analysis recite the efficacy of Tacrolimus treatment in renal transplant patients in association with genetic variants of CYP3A5 gene. Am. J. Clin. Exp. Urol..

[24] van Gelder T., Meziyerh S., Swen J.J., de Vries A.P.J., Moes D.J.A.R. (2020). The Clinical Impact of the C0/D Ratio and the CYP3A5 Genotype on Outcome in Tacrolimus Treated Kidney Transplant Recipients. Front Pharmacol..

[25] Pallet N., Etienne I., Buchler M., Bailly E., Hurault de Ligny B., Choukroun G., Colosio C., Thierry A., Vigneau C., Moulin B. (2016). Long-Term Clinical Impact of Adaptation of Initial Tacrolimus Dosing to CYP3A5 Genotype. Am. J. Transplant..

[26] van Gelder T. (2002). Drug interactions with tacrolimus. Drug. Saf..

[27] Anglicheau D., Flamant M., Schlageter M.H., Martinez F., Cassinat B., Beaune P., Legendre C., Thervet E. (2003). Pharmacokinetic interaction between corticosteroids and tacrolimus after renal transplantation. Nephrol. Dial. Transplant..

[28] Thölking G., Siats L., Fortmann C., Koch R., Hüsing A., Cicinnati V.R., Gerth H.U., Wolters H.H., Anthoni C., Pavenstädt H. (2016). Tacrolimus Concentration/Dose Ratio is Associated with Renal Function After Liver Transplantation. Ann. Transplant..

[29] von Samson-Himmelstjerna F.A., Messtorff M.L., Kakavand N., Eisenberger U., Korth J., Lange U., Kolbrink B., Aldag L., Schulze Dieckhoff T., Feldkamp T. (2023). The Tacrolimus Concentration/Dose Ratio Does Not Predict Early Complications After Kidney Transplantation. Transpl. Int..

[30] Hirsch H.H., Yakhontova K., Lu M., Manzetti J. (2016). BK Polyomavirus Replication in Renal Tubular Epithelial Cells Is Inhibited by Sirolimus, but Activated by Tacrolimus Through a Pathway Involving FKBP-12. Am. J. Transplant..

